# Perceptions of caregivers regarding engagement with integrated management of chronic kidney disease patients in selected public hospitals of KwaZulu-Natal region, South Africa

**DOI:** 10.4102/hsag.v23i0.1104

**Published:** 2018-09-26

**Authors:** Geldine Chironda, Busisiwe Bhengu

**Affiliations:** 1 School of Nursing and Public Health, College of Health Sciences, University of KwaZulu-Natal, South Africa

## Abstract

**Background:**

Chronic kidney disease (CKD) patients rely on non-professional health care providers, namely caregivers to manage their long-term condition. Despite the growing literature on CKD patients, little is known about the perceptions of caregivers regarding integrated management of CKD.

**Aim:**

The aim of the study was to explore the perceptions of caregivers with regard to integrated management of CKD patients.

**Setting:**

The study took place in selected public hospitals of KwaZulu-Natal Province, South Africa.

**Method:**

A qualitative case study design was used. A purposive sampling method was used to select the study participants. Data were collected through a semi-structured interview schedule developed from the literature. Data were analysed through thematic template approach using Health Belief Model constructs.

**Results:**

Hypertension and diabetes mellitus were risk factors that worsen progression of CKD. Unemployment, lifestyle changes and limited social interaction were revealed as negative effects of CKD. Caregivers were aware of consequences of non-engagement with integrated management. The revealed positive benefits of integrated management were mainly physiological and system-related. Barriers to engagement with integrated management were side effects of diet and haemodialysis, hot weather, unemployment, false perception of good health and shortage of kidneys for transplant.

**Conclusion:**

Chronic kidney disease patients require caregivers support to help with necessary changes to cope and adapt with integrated management of the disease. These caregivers experience effects of CKD, consequences of non-engagement and barriers to integrated management. Identification of caregivers perceptions offers healthcare workers a better understanding and formulation of strategies that can offer adequate support to this population.

## Introduction

### Research problem

Chronic kidney disease (CKD) is a slow progressive irreversible deterioration in renal function that results in the kidney’s inability to eliminate waste products, maintain acid base, fluid electrolyte balance and haemopoiesis. The prevalence is estimated to be 8% – 16% in all continents (Jha et al. 2013). In Africa, studies available report a prevalence of about 10% (Barsoum 2013). The prevalence of CKD in South Africa is 14.3% (Duran 2014). Assounga, Hariparshad and Madala (2012) revealed the approximate incidence of CKD from 1000 to 2000 patients per year in KwaZulu-Natal (KZN) Province.

Common causes of CKD are diabetes mellitus and hypertension (Shailendranath, Ushadevi & Prashant 2014; Venkateswararao et al. 2015). Hypertension is the leading cause of CKD in Sub-Saharan Africa and accounts for 45.6% of CKD in South Africa (Assounga et al. 2012). Some other causes include glomerulonephritis, interstitial nephritis, congenital malformations, genetic disorders and obstructive uropathy (Barsoum 2013). Other risk factors associated with the development of CKD in addition to comorbid conditions are herbal ingestion, drug intoxication, obesity, poor water supply and sanitation as well as smoking (Ginawi, Ahmed & Al-hazimi 2014; Naicker 2013).

In addition to being a debilitating and disruptive chronic illness (Moran 2016), the integrated management of CKD is complex and demanding as it entails dialysis, medication, dietary and fluid restriction (Herselman 2008; Morton & Fontaine 2009; Palmer et al. 2015). Diuretics, antihypertensive and anti-diabetics are important, as they have been proved to manage comorbid conditions whilst, at the same time, slowing the progression of CKD to the end of life stages (Morton & Fontaine 2009). Nutritional therapy in CKD forms part of the management. It minimises uremic and anaemia symptoms, reduces the incidence of fluid, electrolyte and acid base imbalances, decrease patient’s vulnerability to infections and limit catabolism (Morton & Fontaine 2009). Removal and control of excess fluid is the cornerstone of volume management in CKD patients (Abraham & George 2016). A more frequent dialysis removes excess fluid, toxins and solutes from blood (Abraham & George 2016) through three principal mechanisms: osmosis, diffusion and filtration (Irwin & Rippe 2012).

Caregivers play a pivotal role as they offer support and this has shown to improve survival and quality of life (Revenson et al. 2016) in CKD patients. Social support of friends and family has been found to influence the integrated management of CKD patients (Karamanidou et al. 2008). However, CKD and its complex integrated management restrict daily activities, employment, family life and social relationships (Harilall & Kasiram 2011; Subhashini & Indira 2016). The burden of care placed on caregivers who need to assume multiple roles that need attention as burnout and compassion fatigue (Mashayekhi, Pilevarzadeh & Rafati 2015), is likely to affect the quality of care for CKD patients. Furthermore, caregiving is associated with difficulties such as depression, a variety of anxiety-related symptoms, a negative impact on perception of physical health and an impaired quality of life (Subhashini & Indira 2016).

According to the South African context, admission of CKD patients to the integrated management programme in public hospitals, entails eligibility for a kidney transplant as dictated by the strict selection criteria (Guidelines for Chronic Renal Dialysis 2009). However, there is insufficient kidney transplantations in public hospitals because of global shortage of organ donations (Bhengu & Uys 2004). It results in limited patient engagement with integrated management (Chironda & Bhengu 2016) with due uncertainty about chances of getting a kidney; thus placing the burden on caregivers. Family members of patients with CKD are struggling to maintain control over their daily lives and have a constant concern about their family member’s condition (Ghai et al. 2015).

Caregivers are in an optimal position to identify challenges of CKD patients in relation to their engagement with integrated management. A review of literature revealed that there is paucity of information about the perceptions of caregivers regarding engagement with integrated management of CKD patients and no studies have examined this concept in KZN Province, South Africa. Hence, this qualitative study was conducted to explore the perceptions of caregivers with regard to engagement with integrated management of CKD patients. Findings of this study may add important information to the literature regarding caregivers’ perceptions on comprehensive management of their respective CKD patients.

The Health Belief Model (HBM) identifies four main beliefs that can influence the management of CKD patients: perceived risk factors, perceived benefits, perceived barriers and severity of outcome of non-adherence (Becker 1974). The model postulates that health-seeking behaviour is influenced by a person’s perception of threat posed by a health problem and the value associated with actions aimed at reducing the threat. In this regard, HBM constructs were used to explore the perceptions of caregivers with regard to engagement with integrated management of CKD patients.

### Purpose of the study

The purpose of the study was to explore the perceptions of caregivers regarding engagement with integrated management of CKD patients in selected public hospitals of KZN Province, South Africa.

### Research question

What are the perceptions of caregivers regarding engagement with integrated management of CKD patients in selected public hospitals of KZN Province, South Africa?

### Definition of concepts

#### Chronic kidney disease

Chronic kidney disease is a slow, progressive irreversible deterioration in renal function that results in the kidney’s inability to eliminate waste products, maintain acid base, fluid and electrolyte balance and haemopoietin. It progresses in five stages (Lowth 2013). In this context, haemodialysis, continuous ambulatory peritoneal dialysis (CAPD) and non-dialysis patients on the CKD programme were all treated as CKD patients.

#### Integrated management

Integrated management is defined as the provision of person-centred treatment and care of chronic patients in which health services work with each other and the patient to ensure coordination, consistency and continuity through different stages of their condition (Integrated Chronic Disease Management 2016). In this study, integrated management refers to provision of CKD comprehensive patient-centred care in terms of dialysis plan, medication, dietary and fluid restriction to give clinically accountable solution focused on enhancing cost-effective and safe management of patients as to slow progression and complications of CKD.

#### Engagement with integrated management

Engagement refers to actions that individuals must take to obtain the greatest benefit from the health services available to them. This focuses on behaviours of individuals relative to their health care that are critical and proximal to health outcomes rather than the actions of professionals or policies of institutions (Centre for Advancing Health Care 2010). In this study, engagement with integrated management is conceptualised as active participation or involvement of caregivers with medication, dialysis, dietary and fluid restriction of their respective CKD patients to prevent complications, manage and promote highest levels of adherent behaviours.

#### Caregivers

Belasco et al. (2006) identified a caregiver as an individual who is closely involved in caring for the patient and, at the same time, helping the patient to cope with prescribed management of the condition. In this study, caregiver is conceptualised as an adult who takes care of CKD patient at home, namely parents or guardians and children.

## Research methods and design

### Research design

A qualitative case study design was used in this study (Yin 2014). Case study design was used to gain in-depth insights on issues of engagement with integrated management from the perspective of selected cases of caregivers.

### Study setting

The study was carried out at two public hospitals in Durban, KZN Province. These hospitals work collaboratively in the provision of integrated management for CKD patients. Moreover, they serve the population of the KZN Province including neighbouring provinces such as Eastern Cape and Mpumalanga. Therefore, focusing on these centres posed the likelihood of capturing caregivers of CKD patients from all three provinces of South Africa.

### Study population

The study population consisted of caregivers of CKD patients attending the selected public hospitals in KZN Province at the time of the study. Caregivers, directly involved in the care of CKD patients at home, were included in the study. Each selected caregiver constituted a case. Furthermore, those who have cared for CKD patients for at least three months were included in the study, as they were able to give realistic perceptions regarding integrated management of CKD patients. Any minors in the form of caregivers were excluded from the study, because they were not able to give informed consent. Those who were not directly involved in day-to-day care basis were also excluded, because they would not able to give realistic perceptions of CKD patients with regard to their integrated management.

### Sample size

A purposive sampling method was used to select cases of caregivers that provided in-depth insights on their perceptions regarding engagement with integrated management of CKD patients. A guiding principle in adequacy of this qualitative case study was data saturation (Grove, Burns & Gray 2013). The researcher started with two cases of caregivers to give in-depth information on their perceptions with integrated management of CKD patients. Four more cases of caregivers were added. Hence, data saturation for this qualitative inquiry was achieved at case number six where no new information was provided by the last selected caregiver.

### Data collection methods

Data were collected using a semi-structured interview schedule, namely perceptions of caregivers regarding engagement with integrated management of CKD patients. Face-to-face method was used to elicit responses from caregivers. The interview scheduled guide was developed from in-depth literature review using constructs of HBM (Rosenstock 1974). The semi-structured interview schedule was designed to elicit in-depth information on perceptions of caregivers with regard to engagement with integrated management of CKD patients. Caregivers were asked on risk factors that worsen progression of CKD, severity of CKD on social life, occupation, family life and performance of activities of daily living, severity of non-engagement of CKD patients with their integrated management, perceived benefits and barriers of CKD patients regarding engagement with their integrated management. The instrument was given to experts, that is, experts in the school of nursing and public health, nephrologists and renal specialists in the clinical area to assess content validity. Permission to carry out the study was sought from the Department of Health, KZN Province, Chief Executive Officer (CEO) for King Edwards and Inkosi Albert Luthuli Hospitals.

### Trustworthiness in qualitative case study

Four measures of trustworthiness were identified for this study: credibility, dependability, confirmability and transferability (Polit & Beck 2012). Credibility was achieved as the researcher spent time with selected cases of caregivers and developed an in-depth understanding of their perceptions during data collection until there was data saturation. A detailed case study protocol with a set of questions to be addressed by the researcher and a data base with field notes, documents and archival records was set in the data collection process so that other researchers can follow the procedures; thus facilitating dependability. Verification of interviews was done with the selected cases of caregivers to confirm that the information collected was a true reflection of their perceptions. The researcher provided detailed descriptive information of the research setting, study participants and themes identified in the study to facilitate assessment for applicability to new situations by prospective researchers in other fields; thus enhancing the transferability of the qualitative case study.

### Data analysis

Data were analysed, using thematic framework in cooperating constructs of HBM (Miles, Huberman & Saldaña 2014; Rosenstock 1974). Data were organised into easily retrievable sections which were according to questions on the interview guide. The researcher familiarised herself with the data by reading through it to search for meanings and patterns until she was familiar with the depth and breadth of the content. Qualitative data were coded and analysed concurrently with data collection. Coding process was used to generate a description of the main and emerging themes for analysis. The main themes were generated, using the constructs of HBM. After coding, the results were summarised thematically to template style analysis including emerging themes. The emerging themes were the ones that appeared as major results in qualitative phase and they were used as subheadings. The emerged themes were made into narrative passages and the findings emerged logically from the caregivers’ responses. Verbatim quotes were provided which the researcher perceived to be most descriptive of the caregivers’ perceptions with regard to engagement of integrated management of CKD patients.

### Ethical considerations

The protocol for research has been approved by the Biomedical Research Ethics Committee (BREC) of the University of KwaZulu-Natal, South Africa (reference number: BE377/2014). Informed consent and participant authorisation were sought from the study participants.

## Findings

### Selected cases of caregivers

Table 1 displays the selected caregivers. Age ranged from a minimum of 20 years to a maximum of 53 years. Of note, a 20-year-old caregiver was looking after her father, because the parents separated long ago. The sample consisted of more female caregivers (five) than male caregivers (one). There were four married and two single caregivers. Most of them (five) had no income and only one was receiving a monthly income of about R6000–R10 000, because he had a job. The majority of participants had no monthly income, because they were not employed and were earlier dependent on the CKD patients before the onset of illness.

**TABLE 1:** Caregivers of chronic kidney disease participants (*N* = 6).

Participant	Age	Gender	Marital status	Occupation	Monthly income
Caregiver 1	20	Female	Single	Unemployed	Nil
Caregiver 2	53	Male	Married	Professional	R6000 – R10 000
Caregiver 3	48	Female	Married	Unemployed	Nil
Caregiver 4	38	Female	Married	Unemployed	Nil
Caregiver 5	44	Female	Married	Unemployed	Nil
Caregiver 6	45	Female	Married	Unemployed	Nil

### Identified themes for selected cases of caregivers

Table 2 reveals the summary of the main and emerging themes from the perspective of caregivers regarding engagement with integrated management of CKD patients. Hypertension and diabetes mellitus were the most highlighted risk factors of CKD confirmed by the caregivers. Unemployment, changes in lifestyle and limited social interaction were cited as negative effects of CKD. Consequences of non-engagement with integrated management included difficulty in breathing, swollen lower and upper limbs, readmissions, abnormal blood results, additional treatment and faster progression of CKD to late stages that require dialysis. The highlighted positive benefits of CKD patients engaging with their integrated management included improved lifespan, less complications, reduced hospitalisations and delayed progression of the disease to late stages and automatic qualification of getting a kidney transplant if one were on the programme. Barriers to engagement with integrated management were side effects of diet and haemodialysis, hot weather, unemployment, false perception of good health and shortage of kidneys for transplantation.

**TABLE 2:** Summary of the main themes and emerging themes from the qualitative case study findings.

Target population	Number of caregivers	Main themes	Emerging themes
Caregivers of CKD patients	6	Risk factors that worsen CKD progression	Hypertension Diabetes mellitus
Caregivers of CKD patients	6	Consequences of CKD	Unemployment Lifestyle changes Limited social interaction
Caregivers of CKD patients	6	Consequences of non-engagement with integrated management	Difficulty in breathing Swollen of the lower and upper limbs Hospital readmissions Abnormal blood results Additional treatment Faster progression of CKD to late stages
Caregivers of CKD patients	6	Benefits of engagement with integrated management	Improved lifespan Less complications Prospects of getting a kidney transplant Reduced hospitalisations Delayed progression of the disease to late stages
Caregivers of CKD patients	6	Barriers to engagement with integrated management of CKD	Side effects of haemodialysis Gastrointestinal problems Stress and false perception of good health Hot weather Shortage of kidneys for transplantation Unemployment

CKD, chronic kidney disease.

### Interrelating and description of themes identified from selected cases of caregivers

The literature was linked to the themes identified and reconstructing interpretations into something meaningful. This process was influenced both by the original research objectives and new concepts generated inductively from the data. The emerged themes were made into narrative passages and the findings emerged logically from the participants’ responses. Verbatim quotes were set as extracts and they were used to give the reader an idea of how main and emerging themes evolved during the process of data analysis in this case study. The main and emerging themes were the following: risk factors that worsen the progression of chronic kidney disease, consequences of chronic kidney disease, consequences of non-engagement with integrated management, benefits of engaging with integrated management and barriers to engagement with integrated management.

#### Risk factors that worsen the progression of chronic kidney disease

In this section, the researcher assessed the caregivers’ perceptions of risk factors that worsen the progression of CKD. All caregivers asserted hypertension and diabetes mellitus as the most common risk factors as expressed by the following excerpt:

‘Anyone with hypertension and diabetes mellitus has higher risk for problems of CKD. My grandfather had hypertension which later caused CKD. Now my father suffered from hypertension for years and then developed CKD’. (Caregiver 1, female 20 years)

#### Consequences of chronic kidney disease

Caregivers cited negative effects of the disease (CKD) on occupation, family life and social relations. Three of the caregivers mentioned that their CKD patients were unemployed, as they were finally boarded off on medical grounds of having the chronic illness after they had been on light duty for almost two years in their companies. This consequently affected their family life, as they were the breadwinners and ended up not fully providing for the families. Lifestyle changes were also highlighted by two caregivers, as the families had to adapt to new dialysis and dietary menus to accommodate CKD patient. Four caregivers verbalised travel restrictions with their CKD patients, as they had to attend dialysis sessions either on daily basis at home or twice a week at the hospital:

‘My father used to work but he was boarded off on medical grounds because of his condition. First, he was given light duties and off sick on days he was attending haemodialysis and this happened for about 1 year. After being dismissed on medical grounds, it affected us financially because there are still other two siblings to finish school’. (Caregiver 1, female, 20 years)

‘Our lifestyle changed when my husband was diagnosed with CKD because we had to adapt to dietary changes as we cannot afford to cook two meals each time we cook at home. Again, sometimes relatives die in rural areas and my husband cannot even go there due to the nature of the illness. I am always representing him as a wife. It is really difficult for other relatives to understand because they do not have knowledge regarding the nature of the disease’. (Caregiver 3, female, 48 years)

#### Consequences of non-engagement with integrated management

All caregivers felt that if a CKD participant does not engage with his or her integrated management, there are severe side effects that will, in turn, affect them. The cited consequences included difficulty in breathing, swollen lower and upper limbs, readmissions, abnormal blood results and additional treatment. One caregiver cited faster progression of CKD to late stages that require dialysis as expressed in the following excerpts:

‘I personally witnessed the consequences of not engaging with dialysis, medication and fluid intake of CKD. My wife was still in denial on initial diagnosis of the disease and she did not attend dialysis sessions for one week. She was drinking fluids unnecessarily not taking into consideration the recommended fluid intake. She started having difficulty in breathing and all the upper and lower limbs were swollen. I had to rush her to hospital for emergency care and she ended being admitted for haemodialysis to normalise the urea in the body’. (Caregiver 2, male, 53 years)

‘My child is still under monitoring and she has been there for 2 years. She has to actively follow her treatment as this will minimise faster progression of the condition to late stages which require dialysis’. (Caregiver 5, female, 44 years)

#### Benefits of engaging with integrated management

In this study, all caregivers believed that there are benefits to CKD patients of engaging with their integrated management. The highlighted positive benefits included improved lifespan, less complications, reduced hospitalisations and delayed progression to late stages of CKD. Moreover, all caregivers highlighted the automatic qualification of getting a kidney transplant for CKD patients on the programme. The following excerpts highlight the above:

‘Of course there are many benefits to CKD patients when they participate in their management. My father is still alive because he is always following his treatment orders. He is also on the transplant list. Since he stated haemodialysis, he has never been admitted to hospital due to complications because he tries to engaging with his treatment regimen though sometimes it’s difficult with diet and fluid’. (Caregiver 1, female, 20 years)

‘My daughter is still in the monitoring phase of CKD and she had tried to follow the recommended treatment and this has delayed the progression of the disease to later stages as she has been on the monitoring for 2 years now. And who knows, she might get a kidney donor soon’. (Caregiver 5, female, 44 year)

**Barriers to engagement with integrated management Side effects of haemodialysis:** Caregiver 2 reported haemodialysis side effects as barriers to haemodialysis for his CKD patient. The barriers cited during haemodialysis were hypotension, severe muscle cramps, nausea and vomiting as expressed in the following excerpt:

‘During haemodialysis, my spouse always had problems like low blood pressure, cramps, nausea and vomiting. When this happens, she does not finish the haemodialysis’. (Caregiver 2, male, 52 year)

**Gastrointestinal problems:** Three caregivers reported gastrointestinal problems as a barrier to engagement with diet among their CKD patients. These included loss of appetite, nausea, vomiting and abdominal pain. The following excerpts reveal the above-mentioned problems by caregivers:

‘My husband always has problems with diet. After eating the renal diet, he complains of nausea first and sometimes he vomits. Abdominal pain follows as well and sometimes diarrhoea’. (Caregiver 4, female, 30 years)

‘My husband is always complaining of loss of appetite because the renal diet is tasteless. Sometimes he feels like vomiting when eating the required food. He ends up eating our food instead of taking his’. (Caregiver 6, female, 45 years)

Hot weather: Hot weather conditions in the study area were cited as a barrier to fluid restriction by all caregivers of CKD patients as expressed in the following excerpts:

‘The most difficult management in patients with CKD is fluid restriction. This is because this area is very hot, so these patients are always thirsty and as such are tempted to drink more fluids to reduce their thirst’. (Caregiver 4, female, 38 years)

‘Fluid restriction is difficult especially if it’s a hot sunny day and this area is very hot’. (Caregiver 5, female, 44 years)

Shortage of kidneys for transplant: All caregivers stated that being in the CKD programme automatically qualifies CKD patients for kidney transplantation. Shortage of kidneys for transplantation was cited as a barrier to engagement with integrated management among CKD patients by all caregivers as expressed in the following excerpts:

‘Since my wife started CKD programme, we have been patiently waiting for the donor but it seems we will wait forever because so many CKD patients are still to get before us. This alone sometimes affects my wife and that’s why she does not actively engage herself in the so-called integrated management because she does not see the need if there is no hope of getting the kidney for transplant in the end’. (Caregiver 2, male, 53 years)

‘Kidneys for donation are scarce and my husband is still waiting for the donor. This thing of waiting sometimes makes him loose hope in his treatment and he ends up not engaging in any of the treatments given’. (Caregiver 6, female, 45 years)

Stress and false perception of good health: Three caregivers highlighted stress and false perception of good health as a barrier to engagement with integrated management among CKD patients. These were expressed in the following excerpts:

‘Though my father tries hard with his medication, haemodialysis, diet and fluid restriction, he sometimes feels depressed about the whole situation. He separated with my mother long back and this sometimes gets to him hard because he sees as if he is a burden to us. When he is in this situation, he does not take his meals normally and can even miss a haemodialysis session’. (Caregiver 1, female, 20 years)

‘My daughter is still urinating and sometimes drinks lots of fluid, not taking into consideration the recommended fluid intake because she perceives herself as stronger and getting better yet her kidney function is deteriorating. Another thing is that she is still young looking for a whole healthy future ahead but this condition sometimes stresses her and she ends up not following the recommended diet with an attitude of saying she will die anyway’. (Caregiver 5, female, 44 years)

**Unemployment:** Three caregivers cited unemployment problems among CKD patients as barriers to engagement with their integrated management, specifically diet as displayed in the following excerpts:

‘My father is not working and neither am I. We are surviving on the medical grant and sometimes hand-outs from other family members. It is sometimes difficult to cook two meals at the same time as we cannot afford, so he sometimes joins whatever we are eating even if it’s not the recommended diet. Renal diet is sometimes expensive to us’. (Caregiver 1, female, 20 years)

‘My husband is not working; he got retrenched due to illness. I am not working as well and this sometimes becomes difficult for us, especially on recommended diet for him’. (Caregiver 4, female, 38 years)

## Discussion of results

The perceptions of caregivers with regard to integrated management of CKD patients were explored, using components of HBM. The structure used to discuss the findings was based on the model; hence, the headings were adopted from the components of HBM.

### Perceived risk factors that worsen the progression of chronic kidney disease

Perceived susceptibility refers to a person’s subjective perception of the risk of acquiring an illness or disease (Becker 1974). The caregivers revealed hypertension and diabetes mellitus as comorbid conditions that worsen the progression of CKD. The study findings are corroborated by the results of Shailendranath et al. (2014), Ginawi et al. (2014) and Naicker (2013) who also cited hypertension and diabetes mellitus as the most common risk factors of CKD. The caregivers did not have knowledge about other risk factors such as alcohol abuse, HIV and AIDS, drug and herbal intoxication that worsen CKD. The more caregivers are knowledgeable about all risk factors that worsen the progression of CKD, the more they are likely to encourage the community and CKD patients to engage with healthy lifestyles and their integrated management.

### Perceived severity of chronic kidney disease and non-engagement with integrated management

Perceived severity refers to a person’s feelings about the seriousness of contracting a disease, leaving the disease untreated and leading to medical and social consequences (Becker 1974). In this study, the researcher analysed the consequences of the disease (CKD) on social life as well as of non-engagement with the required integrated management in CKD population.

#### Social consequences of chronic kidney disease

Caregivers revealed detrimental effects of CKD on occupation, family life and social relations. With regard to occupation, CKD patients were boarded off on medical grounds as cited by the caregivers. As a result, caregivers were affected, because their respective CKD participants were the previous breadwinners. These findings are corroborated by the results of Assounga et al. (2012) and Subhashini and Indira (2016) who explained that the ability of CKD patients to work and lead a productive life is hampered by CKD, as they no longer work and able to provide for their families and this includes the caregivers. This situation is even difficult for single parents with children still to take care (Assounga et al. 2012) and this was verbalised by one of the caregivers in this study.

Lifestyle changes were also highlighted, as the caregivers had to adapt to new dietary and dialysis schedules to accommodate the CKD patients. The study findings are affirmed by Ghai et al. (2015) and Assounga et al. (2012). The caregivers described integrated management of CKD as a limitation on patient lifestyles, as it involves time commitments and frustrations; thus creating a burden for them, as they end up assuming multiple roles needs as affirmed by Mashayekhi et al. (2015).

Caregivers verbalised travel restrictions to other distant places or social gatherings as CKD patients had to attend dialysis sessions either on daily basis at home or twice a week at the hospital. Dialysis patients can only visit for not more than two days unless they are to miss another session of haemodialysis and this negatively impacts engagement as well as social well-being of caregivers. Subhashini and Indira (2016) confirmed the findings of the study. Harilall and Kasiram (2011) further stated that CKD patients find it difficult to integrate the requirements of the dialysis regimen into their daily schedules.

#### Consequences of non-engagement with integrated management

Caregivers highlighted consequences of non-engagement with integrated management of CKD patients. These included pulmonary and peripheral oedema, ineffective breathing, additional treatment and elevated urea and electrolytes. For those who were still under monitoring, non-engagement with their integrated management posed for faster progression to late stages of CKD that require dialysis. This confirms the findings in the literature by Assounga et al. (2012) and Tolkof-Rubin (2008). The highlighted consequences pose problems for caregivers, because they are responsible for offering continual support and emergency medical attention. Burnout and fatigue are likely to occur because of these multiple roles (Mashayekhi et al. 2015); thus affecting care of CKD patients.

### Perceived benefits of engaging with integrated management

Perceived benefits refer to a person’s perception of the effectiveness of various actions available to reduce the threat of illness or disease (Becker 1974). In this study, caregivers believed that there are positive benefits when CKD patients engage with their integrated management. The highlighted positive benefits included improved lifespan, less complications and reduced hospitalisations. These findings are corroborated by the results of Assounga et al. (2012), and Morton and Fontaine (2009). Caregivers highlighted prospects of getting a kidney transplant if one were on CKD programme as affirmed by the strict selection criteria (Guidelines for Chronic Renal Dialysis 2009). Therefore, caregivers play an important role in helping CKD patients to engage with their dialysis, medication, fluid and dietary restriction to minimise complications. This will consequently improve the lifespan of CKD patients, giving them a better chance of getting a kidney transplant.

### Perceived barriers to engagement with integrated management

Perceived barriers refer to a person’s feelings about the obstacles to performing a recommended health action (Becker 1974). The recommended health action in this study is for caregivers to encourage CKD patients to engage with their integrated management. Therefore, the researcher explores the caregivers’ perceptions on barriers to engage with integrated management of CKD patients. These barriers were classified according to physiological, environmental, system-related, psychological and socio-economic factors.

#### Physiological barriers

Physiological barriers in this study were related to gastrointestinal problems because of CKD and side effects of haemodialysis. These findings are similar to studies performed by Assounga et al. (2012). These gastrointestinal problems are caused by uraemia which accumulates as a result of non-engagement of CKD patients with their integrated management. Palmer et al. (2015) highlighted dietary requirements of CKD as complex and challenging for patients because of CKD side effects and this confirms the findings of the study. Herselman (2008) confirmed that attempts to convince patients to actively participate in dietary restriction with medical arguments do not produce desired outcomes, as it is difficult to manage.

Nutritional and dialysis therapy in CKD forms part of the management of the condition and its goal is to minimise uremic and anaemia symptoms, reduce the incidence of fluid, electrolyte and acid base imbalances, decrease patient’s vulnerability to infections and limit catabolism (Morton & Fontaine 2009). However, this goal is hampered by gastrointestinal problems in this study. As a result, there is limited engagement with integrated management among CKD patients, thus adding burden on caregivers.

#### Psychological barriers

Psychological barriers are mind-associated problems and unhealthy thought patterns that keep individuals away from engaging with their integrated management (Marx 2016). Caregivers highlighted psychological issues as barriers to engagement with integrated management among CKD patients. These issues included stress, loss of hope and ignorance. The study findings are corroborated by the results of Assounga et al. (2012) and Subhashini and Indira (2016). Williams (2009) further highlighted irrational thought such as denial, ignorance and false perception of good health as major contributors to poor engagement with treatment regimen among CKD patients. Caregivers are faced with psychological issues of CKD patients. Therefore, caregiving is associated with depression, a variety of anxiety-related symptoms and an impaired quality of life as highlighted by Subhashini and Indira (2016).

#### Socio-economic barriers

Socio-economic status depends on a combination of variables such as occupation and income. Caregivers cited socio-economic problems as barriers to engagement with integrated management among CKD patients. The socio-economic barriers were related to the fact that the caregivers and their respective CKD patients were unemployed. These findings are corroborated by the results of Assounga et al. (2012) and Subhashini and Indira (2016) who cited unemployment as a core stressor among caregivers and CKD patients. However, a study, conducted by Harilall and Kasiram (2011), showed some CKD patients were able to work and remain economically productive with support from health advisors to keep up supporting their caregivers. They were employed, because they were able to integrate their CKD regimen into their work schedules.

#### Environmental barriers

Hot weather in the study area was cited by caregivers as a barrier to fluid restriction among CKD patients. These findings are corroborated by the results of Gerogianni and Babatsikou (2014). However, high levels of thirst could be because of excessive intake of dietary sodium (Assounga et al. 2012), meaning that CKD patients might not be engaged with their dietary restriction guidelines. In this regard, caregivers should be in a position to identify the real reasons for excessive fluid intake among CKD patients.

#### System-related barriers

Shortage of kidneys for transplantation was cited by caregivers as a barrier to engagement with integrated management as patients have to wait for more years to find a donor, causing them to lose hope in their management. Chironda and Bhengu (2016) confirmed the findings of the study and further explained this as a controversial issue surrounding the management of CKD patients. Shortage of kidneys is a challenge (Assounga et al. 2012) which is also contributing to caregivers losing hope and control of their lives.

## Limitations, recommendations for future research and conclusions

### Limitations of the study

- The face-to-face method for data collection might have introduced bias in the following manner:
  ▪ There was interviewer bias through soliciting and interpretation of information from selected cases of caregivers. The researcher was careful not to allow prior knowledge of the diseases condition affect the way the interview was conducted.
  Information bias was present where the selected cases of caregivers might have given information that is considered to be desirable for the researcher to hear, though the information might not be necessarily a true reflection of their perceptions. This was quite possible, because the researcher introduced herself as a health care worker.
- There was selection bias through the use of a hospital-based sample. Selected cases of caregivers consisted of those who could accompany CKD patients to renal clinic and renal unit. Systematic differences could exist in perceptions between those who could and those who could not make it to the renal unit and renal clinic during the study period. Nevertheless, the researcher tried to minimise selection bias through the use of three different caregivers who care for three different classes of CKD, namely haemodialysis, CAPD and non-dialysing classes.

### Recommendations of the study

- Education of caregivers on risk factors that worsen the progression of CKD to the end of life should be emphasised, as all of them were not aware of all the risk factors. There should be continual education of risk factors of CKD for caregivers so that they will encourage quality healthy lifestyles among CKD patients and prevent further incidence of CKD among the healthy population.
- Emphasis and introduction of awareness campaigns for organ donation should be provided to caregivers as they are the ones who stay in the community with the CKD patients. Kidney transplantation is the most effective way of managing and eradicating the CKD and this will ease the burden of caregivers.
- Dietary restriction is an important part of comprehensive management of CKD patients and most of the caregivers verbalised unaffordability of the renal diet because of low socio-economic status. Therefore, the management should advocate for caregivers and liaise with Social Welfare Department to provide extra money for the diet in addition to the monthly medical grant given to the CKD patients.
- Unemployment is a major issue among caregivers. In this view, the management should advocate sheltered employment to improve the socio-economic status of caregivers as well as CKD patients.
- The findings of this study should be implemented and encouraged as this will promote evidence-based practice.

## Conclusion

This article addresses the most important part of the nephrology science. Most of the time, the caregivers and/or guardians of the patients are not included in the care of their family member. Yet, caregivers are subjected to lifestyle changes that affect their physical, social, economic and psychological well-being. They are supposed to make daily decisions about engaging CKD patients in scheduled appointments, taking prescribed medicine, fluid and dietary restrictions as well as managing the symptoms of CKD and other comorbidities. The revealed perceptions of caregivers inform the need to devise strategies that can promote engagement of CKD patients with their comprehensive management.
